# Mechanical damage to green and red lentil seeds

**DOI:** 10.1002/fsn3.480

**Published:** 2017-06-20

**Authors:** Feizollah Shahbazi, Saman Valizade, Ali Dowlatshah

**Affiliations:** ^1^ Lorestan University Faculty of Agriculture Department of Biosystems Engineering Khoram Abad Iran

**Keywords:** Green lentil, harvesting, impact damage, lentil, red lentil

## Abstract

In this research, the breakage susceptibility of two classes of lentil (green and red) was evaluated as affected by impact energy and seed moisture content. The experiments were conducted at impact energies of 0.1, 0.2 and 0.3 J, and moisture contents of 10, 12.5, 15, 17.5, 20 and 25% (wet basis). Results showed that red lentil seeds had more breakage than green seeds and the difference in breakage percentage between green and red lentil seeds was significant at 0.01% level according to analysis of variance (*p < *.01). Percentage breakage of both green and red lentil seeds increased as the energy of impact increased. With increasing the seed moisture content of the both green and red lentils, the breakage percentage of seeds decreased. The average values of seeds breakage green and red lentil seeds varied from 100 to 67.7% and from 100 to 93.1%, respectively, as the seeds moisture content increased from 10 to 25%. The optimum seed moisture at which minimum damage was observed was 17.5% for green lentil and 15% for red lentil. Mathematical relationships composed of lentil seeds moisture content and energy of impact were developed for accurate description of the breakage percentage of green and red lentil seeds under impact loading.

## 1 INTRODUCTION

Lentil (*Lens culinaris* L.) is a high protein (22–34%) pulse crop used primarily for direct human consumption (Tang, Sokhansanj, & Sosulski, 1991). Lentil is best adapted to the cooler temperate or Mediterranean climates zones of the world. Lentil split is an important source of protein in the Middle East and south Asian regions. Lentil seed is classified into two types: Seeds greater than 50 grams per 1,000 seeds (Chilean/large‐seeded) and seeds 45 grams or less per 1,000 seeds (Persian/small‐seeded). The two market classes of lentil are green and red (Saskatchewan Ministry of Agriculture, 2010).

Mechanical damage to seeds is one of the important problems in harvesting and postharvest processing of grain/seed such as lentil seeds. Damaged lentil commands a lower price in the export market and is vulnerable to attack by insects and mold (Shahbazi, Saffar, & Analooei, 2011b). The quality of the grain/seeds is negatively influenced by mechanized harvesting and the postharvest operations. Significant mechanical damages such as: skin rupture, seed fracture, etc, may be exerted to seed by machinery elements during harvesting, transporting, storage and so on. The damage resulted from mechanical interaction between biological material (seed) and machineries material (steel, rubber etc.). Most researchers confirm that seeds damaged mainly occur in the harvesting and transportation processes, where the seeds are subjected and damage by impact forces by machines element (Shahbazi, Sharafi, Moomevandi, & Daneshvar, 2015). The resistance to mechanical damage caused by impact of seeds among other engineering properties plays an important role in the designing and operation of harvesting, handling, threshing and other operation machines for grain/seeds (Shahbazi, Saffar, & Analloei, 2011a). The mechanical damage to seeds caused by impact forces is affected on the factors such as: velocity and energy of impact, seed structure, seed variety, seed moisture content and adjustment of machinery parts in application. Among the above factors, seed moisture content and impact velocity are important factors (Shahbazi et al., 2011a).

The breakage susceptibility due to impact of a grain sample is an indicator of the likelihood of the kernels to break up during handling and transport. It is epically used by researchers, to the quality evaluation of a grain/seed sample in comparison different seed varieties and different systems for seed processing. Researchers confirmed the significant effects of the seed moisture content and the energy of impact on the seed mechanical damage affirm that the seed breakage significantly increases as the energy of impact increases and as the moisture of the seed decrease (Baryeh, 2002; Shahbazi, Valizadeh, & Dolatshaie, 2012). Bourgeois, Moes, and Stobbe (1995) reported that with increase the threshing speed of wheat seeds from 17.5 to 35 m/s, the abnormal percentage seedling increased from 10% to 25%. Khazaei, Shahbazi, Massah, Nikravesh, and Kianmehr (2008) reported that germination loss of wheat seed subjected to 25 to 30 m/s impact velocity was ranged from 12.0% to 20%, depending on the seeds moisture content.

Damage due to impact has been an interesting subject for research, due to the loss in product quality incurred during harvesting, handling and processing. Different impact damage assessment apparatus have been used by researchers to conduct impact tests on agricultural products such as fruits, grain and seeds. Mechanical damage to seeds due to impact have been studied by many researched such as on wheat seeds by Fraczek and Slipek (1998) and Khazaei et al. (2008), on rape seed wheat kernels by Szwed and Lukaszuk (2007), on chickpea seed by Shahbazi (2011), on navy bean by Shahbazi et al. (2011a), on pinto bean by Shahbazi et al. (2011b) and on cowpea seeds by Shahbazi, Dolatshah, and Valizadeh (2014).

There is no information in the literature about the mechanical damage to green and red lentil seeds due to impact in relationship with the energy of impact and seeds moisture content. Therefore, the objective of this study was to investigate the effects of seed moisture content on the breakage susceptibility of green and red lentil seeds under different impact energies.

## 2 MATERIALS AND METHODS

Two lentils genotypes (*Robatt*– green and *Gachsaran*‐red) for the research were obtained from a field in province of Lorestan, Iran. The seed samples at maturity were harvested by hands and cleaned in an air cleaner. In determining the seeds moisture content, whole lentil samples of about 16 g were dried in an air oven at 130°C for 24 hr and the moisture contents were calculated according to ASAE (1988) standard S352.2. The initial moisture content of green and red lentil seeds were 8.46 and 8.79%, wet based, respectively. The samples with higher moisture content were prepared by spraying predetermined amount of distilled water to specific amount of seeds, then sealing in plastic zip bags and stored at about 5°C for 2 weeks. Before starting each test, the samples were warmed to room temperature and their moisture was measured.

The impact test device use to apply impact to seed samples was similar to impact test devices used by Kim, Opara, Hampton, Hardacre, and MacKay (2002), Shahbazi et al. (2012), Shahbazi (2013), Shahbazi et al. (2014) and Shahbazi (2014). An aluminum drop bar was inserted into a steel tube had 4 mm diameter holes from 5 to 60 cm with 5 cm intervals. The drop height of the aluminum bar, based on the impact energy required, was manually adjusted and controlled by a pin inserted into the hole in the middle of the steel tube. When the pin was manually removed at the given drop height, the aluminum bar dropped, hitting the seed at the base plate. The energy of impact on seed depends on the drop height and the mass of the aluminum bar.

In the current study, the effects of impact energy (0.1, 0.2 and 0.3 J) and lentil seeds moisture content (10, 12.5, 15, 17.5, 20 and 25%, w.b) on the mechanical damage to lentil seeds (percentage seeds breakage) were studied. The rage of the seeds moisture considered from 10 to 25% includes the moisture levels during harvest and postharvest operations for agricultural seeds (Khazaei et al., 2008). The factorial experiment as a completely randomized design with three replicates was conducted. For each impact test, 100 seeds were randomly selected from lentil seed samples and subjected to the impact load by impact test apparatus. After the test, external damage of lentil seeds including the cracked, bruised and broken seeds were removed from samples. The percentage damage to seed was calculated by the following relationship (Shahbazi et al., 2014):

(1) $$\text{Seed Damage}\left(SD\right)=\frac{\text{Weight of damaged seeds}}{\text{(Weight of total seeds (damaged + undamaged))}}\times 100$$

The test data were analyzed by the analysis of variance (ANOVA). The means were compared at the confidence interval of 95%, using Duncan's multiple range test in SPSS 19 software. The nonlinear regression of SAS program (SAS, 1985) was used to fit and find the best models to experimental data and developing modes for the relationships between the percentage damage to the lentil seeds and experimental variables.

## 3 RESULTS AND DISCUSSION

There was a significant difference in breakage percentage between the two classes of lentil (green and red). Both the experimental variables include impact energy and seeds moisture content had a significant effect on the mechanical damage to lentil seeds (Table 1). The impact energy had the most influence (F = 131.83) but the lentil variety (F = 46.81) and the seed moisture content (F = 13.64) had the least, respectively, within the studied boundary for the variables (Table 1). Also the interaction effects of lentil class × seed moisture content, lentil class × impact energy, moisture content × impact energy and the interaction effect of three independent variables significantly influenced the percentage mechanical damage to lentil seeds at the 1% probability level (Table 1).

**Table 1:** The result of the variance analysis of the mechanical damage to lentil seeds as affected by lentil seed class, seeds moisture content, and the energy of impact

Source	DF	Mean square	F
Lentil* *Class (LC)	1	5186.49	46.81^a^
Moisture Content (MC)	5	1511.61	13.64^a^
LC × MC	5	565.398	5.10^a^
Impact Energy (IE)	2	14606.78	131.83^a^
LC × IE	2	3908.03	35.27^a^
MC × IE	10	1024.05	9.24^a^
LC × MC × IE	10	602.36	5.44^a^
Error	72	110.81	

a Significant at the 1% probability level.

**Figure 1:**
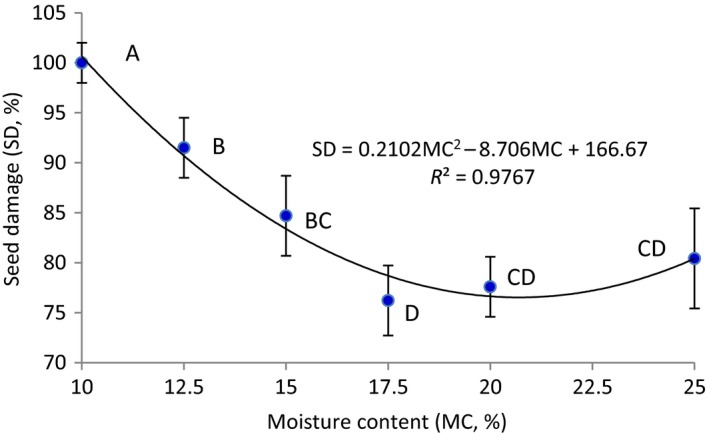
Mechanical damage to lentil seeds (green and red) as affected by moisture content. (Averages with the same letter have no significant difference at the 0.05 level)

Lentil seeds moisture content had high effect on the mechanical damage due to impact loading. The percentage damage of lentil seeds (both green and red) decreased with the increase in the moisture content (Figure 1). These results show that the moisture content of the seeds has significant effects on their elastic properties. With increasing moisture content of seeds their elasticity and firmness will increase which causes a greater absorption of impact energy during impact loading and thereby increases the resistance to impact damage (Evans, Holmes, & McDonald, 1990). On the other hand, at the lower moisture content, the seeds are more brittle, thus, are more prone to mechanical damage under impact loading. Similar results about decreasing for mechanical damage of seeds by increasing their moisture content are reported for rapeseed and wheat by Szwed and Lukaszuk (2007), for wheat seed by Khazaei et al. (2008) and for chickpea seed by Shahbazi (2011). With the increase of moisture content of lentil seed from 10% to 17.5%, the mean values of seeds damage in Figure 1decreased significantly from 100% to 76.2%, however, further increase in seed moisture content from 17.5% to 25%, did not show a significant increase. The average values for the breakage percentage were 100, 91.5, 84.7, 76.2, 77.6 and 80.4% for moisture contents of 10, 12.5, 15, 17.5, 20 and 25% (w.b.), respectively (Figure 1). It is evident from the data in Figure 1, that with the increase in seeds moisture content, the percentage mechanical damage to lentil decreased as a quadratic function. Similar results have been reported by many researchers in other crops (Khazaei et al., 2008; Parde, Kausalb, Jayasa, & White, 2002; Szwed & Lukaszuk, 2007; Tang et al., 1991). Using nonlinear regression techniques, the following relationship between lentil (green and red) seed damage (SD, %) and moisture content (MC, %):

(2) $$SD=166.67-8.706MC+0.2102MC^{2}\;\;R^{2}=0.9767$$

The above equation applies to moisture contents of the following: 10–25%. All the indexes are significant at the level of 99.95%.

**Figure 2:**
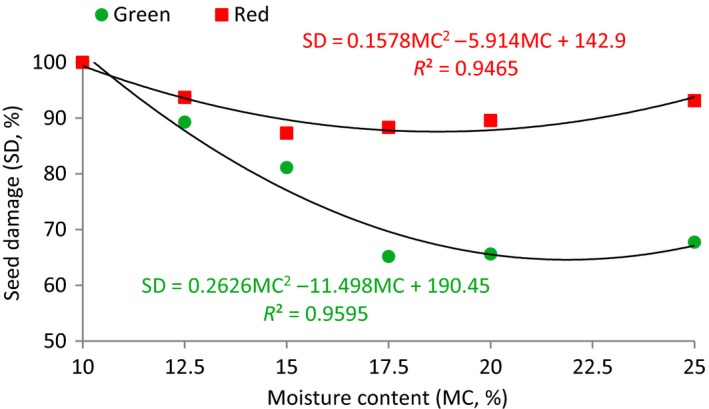
Percentage damage of green and red lentil seeds at different moisture contents

Figure 2 shows the relationship between the mechanical damage to the green and red lentil seeds. In general, the percent of broken seeds, of both lentil classes, increased as seed moisture contents decreased. Percentage damage in red lentil was higher than green lentil at the same seed moisture content studied in the present research.

The average value of the breakage percentage of green lentil at different test conditions in Figure 2 was obtained 78.15% (data not shown) while its value for red lentil seeds was obtained as 92.00%. These results show that red lentil seeds are more brittle than green lentil seeds. The average values for the breakage percentage of green lentil seeds, in Figure 2, were found to be 100, 89.3, 81.1, 65.2, 65.6, and 67.7% (with mean value of 78.1%) at seed moisture contents of 10, 12.5, 15, 17.5, 20, and 25% (w.b.), respectively. The average values for the breakage percentage of red lentil seeds, were found to be 100, 93.7, 87.3, 88.3, 89.6 and 93.1% (with mean value of 92.0%) for same moisture contents.

In Figure 2, the regression equation that was obtained for the relationship between mechanical damage to green lentil seeds (SD, %) and moisture content (MC, %) has an *R* ^*2*^value of 0.9595 (*R* ^*2* ^= 0.9595). The slope of the curve distinctly changes at about 17.5% moisture content, with little breakage above and greatly increasing breakage below this moisture level, indicating that the optimal harvesting, the handling and the processing of green lentil seeds would be about the moisture level of 17.5%. According to several researches, under the effect of impact loading for each seed variety, there is special moisture content at which there occurs a minimum mechanical damage (breakage) to the seeds (Shahbazi et al., 2015). So in this research, the optimum level of moisture content for green lentil seed is about 17.5%. In Figure 2, the exponential relationship between the mechanical damage to red lentil seeds and the seeds moisture content has the *R* ^*2*^ value of .9465. The slope of the curve for the mechanical damage to red lentil seeds decreased at the moisture content of about 15%, showing that the optimum moisture for harvesting and postharvest operation processing for red lentil seeds would be about the moisture content of 15%.

The dependency of seed damage of lentil seed (SD, %) on moisture content (MC, %) was expressed by the following best‐fit equations for green and red verities, respectively:

(3) $$SD=190.45-11.498MC+0.262MC^{2}\;\;R^{2}=0.9595\;\text{for green lentil seeds}$$

(4) $$SD=142.9-5.914MC+0.1578MC^{2}\;\;R^{2}=0.9465\;\text{for red lentil seeds}$$

The above equations apply to seed moisture content about 10–25%. All the indexes are significant at the level of 99.95%.

**Figure 3:**
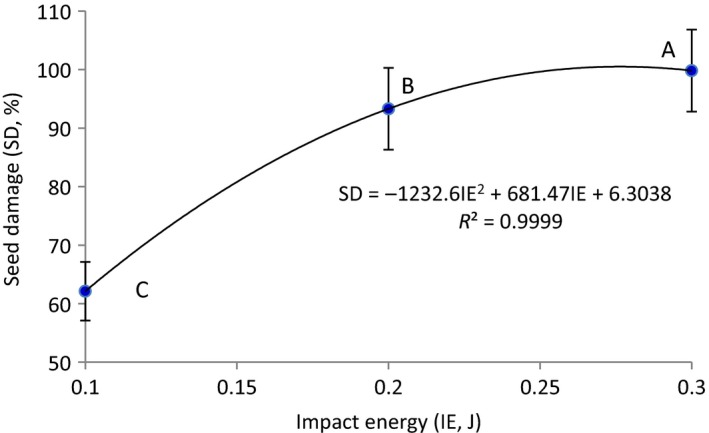
Percentage damage of lentil seeds at different impact energies. Mean values with the same letter are significantly different at the 0.05 level (*p* < .05)

The impact energy had significant effect on the mechanical damage to the both green and red lentil seeds. Figure 3 shows percentage damage of lentil seeds at different impact energies. As shown in Figure 3, lentil seeds mechanical damage increased as a quadratic relationship with increasing in the energy of impact. The mean values of the mechanical damage to lentil seeds at all levels of impact energies are significantly different (*p* = .05) (Figure 3). The mechanical damage to lentil seeds increased by 37.7% by increasing the energy of impact from 0.1 to 0.3 J. The corresponding value for increasing the impact energy from 0.1 to 0.2 J and from 0.2 to 0.3 J, were about 31.16 and 6.52%, respectively. Similar results regarding seed damage due to impact energy have been reported by Shahbazi et al. (2011b) for pinto beans and Shahbazi et al. (2014) for cowpea seeds. Shahbazi et al. (2012) reported that mechanical damage to triticale and wheat seeds increased from 44.8% to 71.6% and from 18.7% to 35.2%, respectively, as the energy of impact increased from 0.05 to 0.1 J.

The lentil seed damage (SD, %) was related to impact energy (IE, J) by the following best‐fit regression equation:

(5) $$SD=6.3038+681.47IE-1232.6.IE^{2}\;\;R^{2}=9999$$

The above equation applies to impact energies 0.1 to 0.3 J. All the indexes are significant at the level of 99.95%.

**Figure 4:**
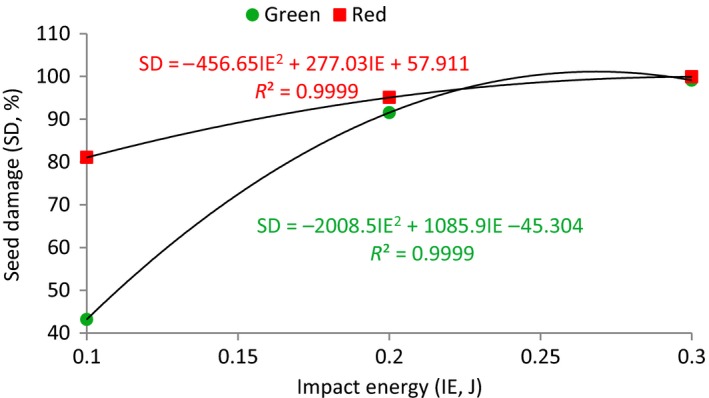
Percentage damage of green and red lentil seeds at different impact energies

Figure 4 shows the percentage damage to green and red lentil seeds at different impact energies. As shown, the percentage damage to both lentil classes seeds increased as the energy of impact increased. But, due to the significant interaction effect between the energy of impact and lentil class, the increasing rates in damage to seeds are not same for both classes. The effect of impact energy on the damage was greater for green lentil seeds than for red lentil seeds.

The lowest breakage percentage was 43.2% occurred at 0.01 J energy of impact with the green lentil seeds while greatest breakage percentage was obtained as 99.9%, occurred at impact energy of 0.3 J with red lentil seeds. By increasing the energy of impact from 0.1 to 0.3 J, the percentage mechanical damage to green and red lentil seeds increased from 43.9 to 100% and from 81.0 to 99.92%, respectively.

The mechanical damage to the green and red lentil seeds was related to the energy of impact in the range of 0.1 to 0.3 J. The results showed that, for two studied lentil classes considered, the percentage damage to seeds was a quadratic equation of the energy of impact.

The model showing the relationship between the percentage seed damage (SD, %) and impact energy (IE, J) for the tow lentil class was presented by the following best‐fit regression equations:

(6) $$SD=-45.304+1085.9IE-2008.5IE^{2}\;\;R^{2}=0.9999\;\text{for green lentil seeds}$$

(7) $$SD=57.911+277.03IE-456.65.IE^{2}\;\;R^{2}=0.9999\;\text{for red lentil seeds}$$

The above equations apply to impact energies 0.1 to 0.3 J. All the indexes are significant at the level of 99.95%.

Based on the above results, for harvest and postharvest operations for lentil seeds, in which seeds are subjected to impact forces, the best conditions for the seed moisture content is about 17.5% and 15% for green and red lentil seeds, respectively, with an impact energy limited to <0.1 J. These features may be considered in the designing and adjusting of the machines for harvest and postharvest operation for lentil seeds, to limit the energy of impact of machinery elements to 0.1 J, selecting the time of harvesting to conditioned seeds to the optimum moisture content, and in minimizing the crop quality losses due to mechanical damaged seeds.

## 4 CONCLUSIONS

Mechanical injury of lentil seeds was genotype dependent and there was a significant difference between the percentage breakage of green and red lentil seeds, where red lentil seeds displayed more breakage than green seeds. The breakage percentage of both green and red lentil seeds was increased as impact energy increased. By increasing the energy of impact from 0.1 to 0.3 J, the percentage mechanical damage to green and red lentil seeds increased from 43.9% to 100% and from 81.0% to 99.92%, respectively. The results showed that an increase in moisture content of both green and red lentil seeds led to a decrease in the percentage damage of seeds as a quadratic function. The average percentage damage to green lentil seeds decreased from 100% to 67.8% as the moisture content increased from 10% to 25%. The percentage damage to red lentil seeds varied from 100% to 93.1% over the above same moisture range. There was an optimum moisture levels about 17.5% and 15%, for green and red lentil seeds, respectively, at which there occurs a minimum mechanical damage (breakage) to the seeds. Mathematical models composed of seed moisture content and impact energy were developed for accurately describing the mechanical damage to lentil seeds under impact loading.

## CONFLICT OF INTEREST

None declared.
